# A molecular perspective of mammalian autophagosome biogenesis

**DOI:** 10.1074/jbc.R117.810366

**Published:** 2018-01-25

**Authors:** Thomas J. Mercer, Andrea Gubas, Sharon A. Tooze

**Affiliations:** From the Molecular Cell Biology of Autophagy Laboratory, The Francis Crick Institute, 1 Midland Road, London NW1 1AT, United Kingdom

**Keywords:** autophagy, intracellular trafficking, phosphatidylinositide 3-kinase (PI 3-kinase), post-translational modification, signaling, ATG proteins, ATG9, ULK1, VPS34, WIPI2

## Abstract

Autophagy is a highly conserved process and is essential for the maintenance of cellular homeostasis. Autophagy occurs at a basal level in all cells, but it can be up-regulated during stress, starvation, or infection. Misregulation of autophagy has been linked to various disorders, including cancer, neurodegeneration, and immune diseases. Here, we discuss the essential proteins acting in the formation of an autophagosome, with a focus on the ULK and VPS34 kinase complexes, phosphatidylinositol 3-phosphate effector proteins, and the transmembrane autophagy-related protein ATG9. The function and regulation of these and other autophagy-related proteins acting during formation will be addressed, in particular during amino acid starvation.

## Introduction

**Figure 1.**
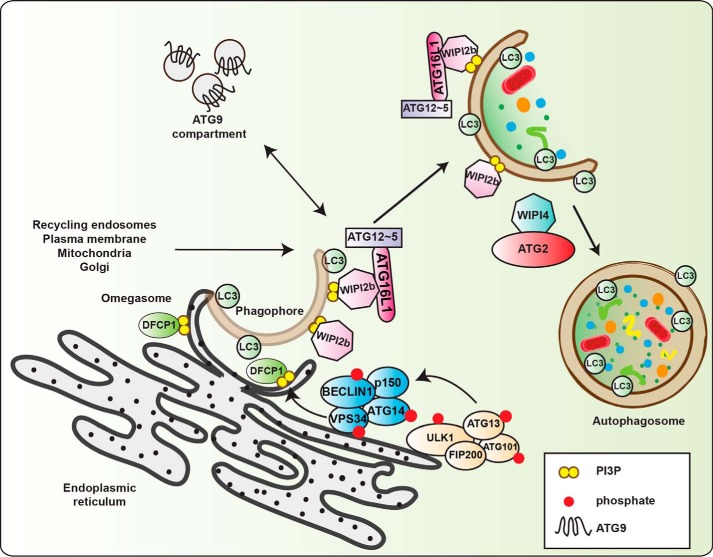
**Process of autophagosome formation.** First steps require the translocation of active ULK1 to omegasomes. ULK1 subsequently activates omegasome-bound VPS34, which generates pools of PI3P at omegasomes. Note for clarity the ULK complex and PI3K complex 1 are shown adjacent to the ER. DFCP1 and WIPI2b bind PI3P, and the latter then recruits the ATG12∼ATG5–ATG16L1 complex enabling LC3 lipidation to the phagophore. Phagophore initiation and elongation are facilitated by transient interactions with the ATG9 compartment, which potentially delivers lipids for membrane formation from sources such as recycling endosomes, plasma membrane, mitochondria, ER, ERGIC, or Golgi. Phagophore growth and closure have been suggested to be controlled by the ATG2–WIPI4 complex.

**Figure 2.**
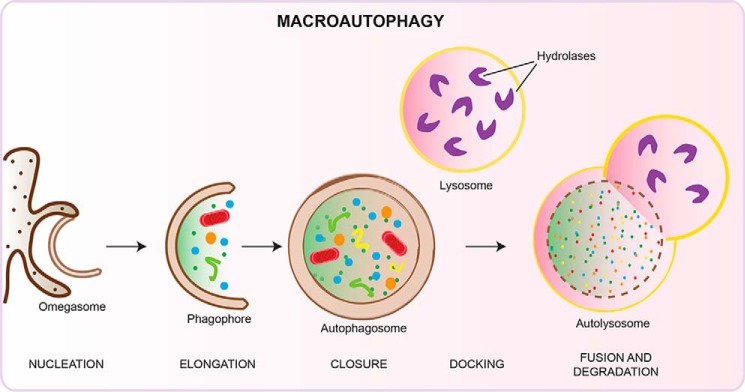
**Schematic depicting the stages of autophagosome formation.** Upon translocation to ER puncta, the autophagy initiation complexes stimulate the formation of an omegasome. This cup-shaped signaling platform recruits autophagy effectors, leading to the production of a double-membraned phagophore. Cargo sequestration occurs concomitantly with phagophore elongation. The phagophore seals forming an autophagosome between 0.5 and 1.5 μm in diameter, which fuses with the lysosome, forming an autolysosome and resulting in the degradation of its contents.

Autophagy is a lysosome-mediated process by which cells recycle cytosolic cargo. A number of stressors are able to stimulate the pathway; however, the best understood stimulus for autophagy is amino acid starvation. Upon stimulation by upstream nutrient/energy-sensing kinases, the ULK1/2 kinase complex becomes active and translocates to ER^2^ puncta followed by the autophagic phosphoinositide 3-kinase (PI3K) complex I. Colocalization of these initiating complexes at the ER leads to the production of PI3P and the recruitment of autophagy effectors to form the omegasome. One of these effectors, WIPI2b, promotes lipidation of LC3B (with this form commonly called LC3-II), one of the members of the Atg8 family of proteins (LC3A/B/C, GABARAP, and GABARAPL1/L2) via two autophagy-specific ubiquitin-like conjugation systems. Driven by insertion of lipidated Atg8 proteins and regulation by distal membrane compartments, including ATG9 vesicles, the phagophore expands, enclosing a portion of the cytosol, and closes to form the autophagosome. Several different subcellular compartments have been suggested to supply membrane to forming phagophores, including the ER, ER-Golgi intermediate compartment (ERGIC), plasma membrane, Golgi, mitochondria, and recycling endosomes (Fig. 1). Finally, the contents of the autophagosome are degraded upon fusion with the lysosome (Fig. 2).

Work with *Saccharomyces cerevisiae* has provided us with much of the initial insight into the mechanisms of autophagy, most notably leading to the discovery of the autophagy-related (ATG) genes. However, this Minireview will cover the molecular machinery involved in mammalian autophagosome biogenesis with a focus on how molecules, membranes, and signaling cascades synergize to regulate each stage.

## ULK complex

The ubiquitously expressed kinases ULK1 and ULK2 carry out a range of catalytic and non-catalytic functions to regulate autophagosome formation from initiation to maturation (1–3). They occupy the most upstream position in the autophagic signaling pathway and are considered the master regulators of autophagy. Expression of kinase-inactive mutants of ULK1 is associated with dominant-negative inhibition of autophagy, with the attainable rate of autophagic flux corresponding to the remaining kinase activity (3, 4). ULK deficiency *in vivo* results in the abrogation of starvation-induced autophagy, along with activation of a misregulated unfolded protein response in neurons (5) and systemic defects via aberrant reactive oxygen species (ROS) neutralization in erythrocytes (6). Although specific roles for ULK1 and ULK2 have been identified for some cell types *in vivo* (7), ULK1 is by far the best characterized and will be the focus of this Minireview.

ULK1 is the only catalytically active component in a heterotetrameric complex with FIP200, ATG13, and ATG101. Both ATG13 and FIP200 stabilize ULK1, increase its kinase activity, and encourage translocalization from the cytosol to the omegasome (8–10). ATG101 helps maintain ULK1 basal phosphorylation and promotes its stabilization along with that of ATG13 (11, 12). Formation of the ULK complex is not regulated by nutrient status (8); however, complex-independent roles for ULK1, FIP200, and ATG13 have been reported (13, 14), indicating that viewing the ULK complex proteins functioning only as a complex may be overly simplistic.

## Sensing the signal

ULK1 is regulated by the nutrient/energy-sensing kinases MTORC1 (mechanistic target of rapamycin complex 1, MTOR herein) and AMP-activated protein kinase (AMPK). MTOR is active in nutrient-replete conditions; it binds ULK1 directly via its RAPTOR subunit in a manner dependent on amino acid availability but independent of ULK activation status (8, 15, 16), and it inhibits autophagy via phosphorylation of ULK1 at serine 638 and 758 and of ATG13 at serine 258 (17–19). Interestingly, an alignment of all phosphorylation sites in ULK1's intrinsically disordered region (known to be extensively phosphorylated) revealed a consensus logo similar to MTOR's, potentially implicating it as a primary phosphoregulator of ULK1 (20). The autophagy-promoting AMPK becomes active upon the depletion of ATP and negatively regulates MTOR activity through phosphorylation of RAPTOR and TSC2 (21, 22). AMPK also binds ULK1 directly (19, 23, 24) leading to the phosphorylation of both ULK1 and ATG13 (17). The AMPK sites in ULK1 include Ser-555, Ser-637, and Thr-659 (Ser-556, Ser-638, and Thr-660 in human ULK1), which among other mechanisms promotes the proper trafficking of ATG9 (24). Serine 638, a substrate for both AMPK and MTOR, is also a target for at least two phosphatases, with both PP2A (25) and PPM1D (26) implicated in autophagy.

In addition to phosphorylation, the role of ubiquitin signaling in ULK1-regulated autophagy is becoming increasingly clear. AMBRA1–TRAF6-dependent Lys-63-linked ubiquitination of ULK1 promotes its dimerization and activation (27). Furthermore, the chaperone protein p32 binds ULK1 and, acting by an unknown E3 ligase, inhibits Lys-48-linked ubiquitination while driving Lys-63-linked ubiquitination. This promotes ULK1 stability and is crucial in both starvation-induced autophagy and mitophagy (28), the selective removal of mitochondria. In another context, ubiquitination negatively regulates ULK1 signaling (29–31). Upon starvation, the E3 ligases NEDD4L and CULLIN3 control the amplitude and duration of the autophagic response by driving ULK1 degradation via Lys-27–linked, Lys-29–linked (NEDD4L), or Lys-48–linked (CULLIN3) ubiquitination (30, 31).

## Relaying the signal

### Membrane association

The ULK complex is mostly cytosolic, although pools exist on recycling endosomes (32), mitochondria (33, 34), and the ER. Upon amino acid starvation, the ULK complex translocates to subdomains of the ER to drive the nucleation of autophagosomes. These regions are shown to coincide with ER–mitochondria contact sites (35) and autophagy-specific ER exit sites, which are specified by ATG9 vesicles (36). The ULK complex is retained at these regions of the ER, called omegasomes, until formation of the phagophore, when it (the ULK complex) is recycled to the cytoplasm (37).

ULK complex anchoring to the omegasome may be facilitated by membrane association of the C-terminal domain or EAT domain (which occurs independent of kinase activity (2, 3, 38)) and ATG13, which directly associates with acidic phospholipids in the membrane via a basic patch in its N terminus (37). Phosphatidylethanolamine-conjugated Atg8 family proteins, which are incorporated into the phagophore, bind ULK1 and ATG13 to further increase their retention at the phagophore (39, 40), which promotes ULK1 kinase activity via a positive feedback loop involving the PI3K complex I (41).

The translocation of ULK1 to the ER also requires a range of protein machinery. For example, the Rab1 effector C9orf72 binds the ULK complex to promote trafficking to the phagophore as well as its activation (42, 43). However, C9orf72 possesses multiple roles in autophagy as, together with its binding partner SMCR8, it has been implicated in autophagosome maturation (44), selective autophagy (45), and both positive and negative regulation of autophagy via modulation of the MTOR/ULK signaling axis (42, 46). Interestingly, recent data suggest unique machinery regulate recruitment of ULK1 to ER–mitochondria contact sites dependent on the stimuli (47); it remains to be established whether this translates to stimulus-specific spatial regulation of ULK1 on the ER.

### ULK substrates

The identification of ULK substrates is crucial if we are to understand the mechanisms of autophagy. Although not essential for ULK1 phosphorylation, many of the targets identified to date associate physically with the ULK complex (Table 1). For example, ULK1 inhibits the AMPK and MTOR complexes by multisite phosphorylation, and three of five members of the PI3K complex 1 are ULK substrates, and ULK1 sites have been identified in SMCR8 (Table 1) (45, 48–50).

**Table 1:**
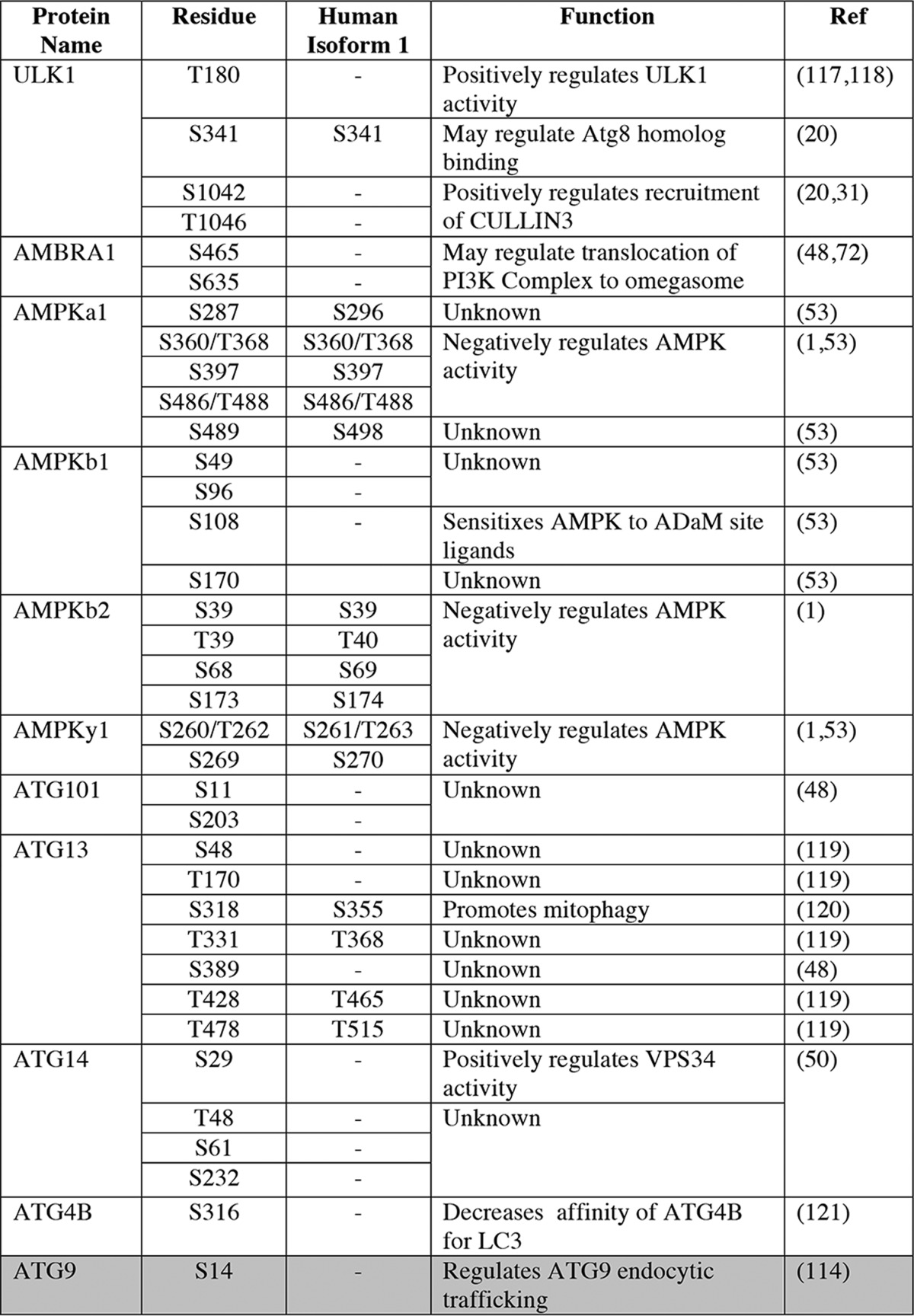

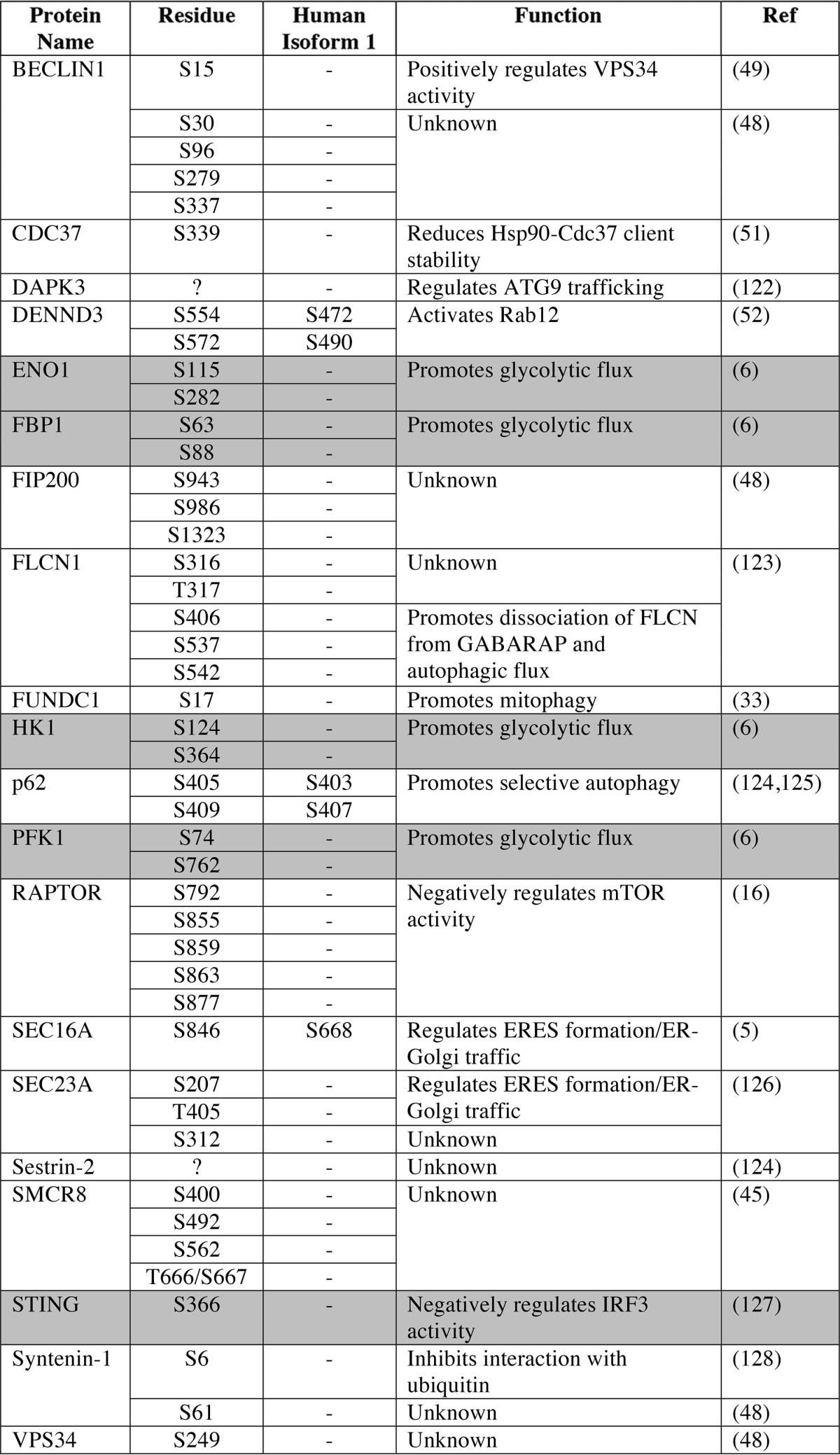
**ULK1 substrates** ULK substrates that are not known to physically interact with the ULK complex are highlighted in gray. The residue identified and the corresponding amino acid in the UniProt designated human isoform 1 are listed, followed by the proposed function of each phosphorylation event where the data are available. Where the exact phosphoacceptor residue is not known, the potential residues are listed separated by a dash. Notably, the function of many of the phosphorylation events listed is uncertain. Refs. 120–128 are cited in the table.

A recently published consensus motif has provided an important tool for the identification of ULK substrates *in vivo* (48). ULK1 was found to possess selectivity for aromatic hydrophobics at −3 and aliphatic hydrophobics/serine at positions +1/2 relative to the phosphoserine (48). Importantly, however, a number of previously identified ULK substrates do not conform to the consensus motif (1, 6, 16, 20, 31, 45, 48, 50–56), demonstrating that our understanding of what constitutes an ULK site remains incomplete.

## PI3K complex

Immediately downstream of the ULK complex is the primordial class III PI3K VPS34 (PIK3C3). VPS34 translocates to ER puncta soon after ULK, where it produces a pool of PI3P to drive omegasome formation (37). VPS34 activity is crucial for starvation-induced and basal autophagy (57–59) and conversely for MTOR activation upon amino acid sensing (60).

VPS34 never works alone. The PI3K core complex comprises VPS34, beclin 1 (BECN1), and the pseudokinase p150 (PIK3R4). Depending on the subcellular context, the core complex binds ATG14 or UVRAG in a mutually exclusive manner defining the PI3K complexes 1 and 2, respectively (61). Furthermore, a significant proportion of VPS34 is found in a binary subcomplex with p150, with the relative abundance of each complex being cell type–specific (61). Removal of individual components results in the depletion of other VPS34 complex proteins, significantly hampering starvation-induced autophagy and resulting in early embryonic lethality (62–66).

Alongside the non-autophagic roles in endomembrane trafficking and multivesicular body formation (67), the UVRAG-containing PI3K complex 2 is primarily implicated in the later stages of autophagy, such as autophagosome–lysosome fusion and the scission of autolysosomal tubules (68, 69). Meanwhile, the autophagy-specific ATG14-containing PI3K complex 1 positively regulates autophagosome nucleation and will be the focus of this section.

## Association of PI3K complex 1 with autophagic membranes

A number of physical properties of the PI3K complex 1 promote its association with membranes. An aromatic finger in BECN1 (70) and N-terminal myristate on p150 (71) ensure that PI3K core complex remains tightly bound to lipid bilayers. ATG14 brings with it ER-specific targeting motifs, including an N-terminal cysteine-rich domain, which is not actively regulated (72), and the BATS domain, which binds PI3P and phosphatidylinositol 4,5-bisphosphate to promote recruitment to curved membranes and complex stability, respectively (73, 74).

In basal conditions, a pool of VPS34 is tethered to the cytoskeleton via an association between BECN1-interactor AMBRA1 and microtubules, which is disrupted upon starvation to allow translocation to omegasomes (75). Another component involved is the multi-membrane spanning protein VMP1 (76). VMP1 localizes to punctate ER membrane contact sites (77) and binds BECN1 to exclude it from its antagonist BCl2 (78). Complex 1 association is further stabilized by direct binding of ATG14 and ATG13 (50). Interestingly, another ER-resident protein, STX17, also recruits ATG14 to ER–mitochondria contact sites to drive autophagosome biogenesis in a starvation-dependent manner (35).

## Regulation of PI3K complex 1 activity

A host of regulatory proteins impinge on the autophagy pathway through association with or modification of the PI3K complex. Notably NRBF2 (79–81), Dapper1 (59), PAQR3 (82), and RACK1 (58) all stabilize VPS34 and promote autophagy via association with the ATG14–BECN1 subcomplex. NRBF2, which was identified as an interactor of ULK1 (83), can both promote (80, 81) or inhibit (84) VPS34 activity depending on the cellular context (85). Recently, NRBF2 was shown to facilitate dimerization of PI3K complex 1 heterotetramers, which occurs independently of kinase modulation (80).

A large number of phosphorylation sites have been identified in VPS34 complex 1 components and regulatory proteins. As covered in Table 1, ULK1 phosphorylates VPS34, BECN1, and ATG14, with two of these phosphorylation sites (BECN1 Ser-15 and ATG14 Ser-29) known to promote omegasome formation and VPS34 activity *in vitro* and *in vivo* (49, 50). Furthermore, the previously mentioned starvation-dependent release of VPS34 from microtubules relies on phosphorylation of AMBRA1 by ULK1 (75).

In addition to ULK1, other kinases implicated in the stress response impinge on autophagy through regulation of VPS34 activity. Phosphorylation of NRBF2 by MTOR switches it from a positive to a negative regulator of complex 1 activity (85). Five inhibitory MTOR phosphorylation sites were identified in ATG14 (86), and the aforementioned positive regulation of ULK1 via TRAF6-dependent ubiquitination relies on the dephosphorylation of serine 52 in AMBRA1, an MTOR target site (27). Direct phosphorylation of VPS34 by AMPK inhibits PI3P production in the absence of ATG14/UVRAG (61). However, AMPK also phosphorylates BECN1 at serines 91 and 94 (serines 90 and 93 in the human protein), which activates VPS34 activity *in vivo* and is facilitated by ATG14 (61) and at threonine 388, which enhances both PI3K complex 1 formation and autophagic flux (87). Furthermore, AMPK must phosphorylate RACK before it is able to scaffold complex 1 formation (58) and PAQR3 for it to augment VPS34 activation on glucose starvation (82). The association of mitogen-activated protein kinase signaling components with BECN1 also occurs upon autophagy activation (88), with MAPKAPK2 directly phosphorylating BECN1, also at Ser-90, to promote autophagy during nutrient depletion (89). Two more phosphoregulatory enzymes are known to act at Ser-90, DAPK3, and the previously mentioned phosphatase PP2A (90).

A range of post-translational modifications govern VPS34 function in autophagy initiation. Like ULK1, the PI3K complex 1 is actively regulated by ubiquitination in a starvation-dependent manner, which, depending on the context, regulates either its stability or activity to modulate autophagy (31, 91–94). Of note, the deubiquitinating protein ataxin-3 (ATXN3) was recently shown to bind BECN1 via a polyglutamine tract, promoting its stability and consequently starvation-induced autophagy. BECN1 was competitively displaced from ATXN3 in the presence of the exogenously expressed polyglutamine domain from huntingtin, the protein implicated in Huntington's disease, potentially revealing a mechanism for the autophagy defect in polyglutamine diseases (91).

Once activated at the cytosolic face of the ER, VPS34 phosphorylates phosphatidylinositol to produce PI3P. In basal conditions, very little PI3P is present on the ER (95). Production of this charged signaling lipid leads to recruitment of effectors, ultimately culminating in the membrane rearrangements associated with autophagosome biogenesis.

## PI3P effectors

### FYVE-containing proteins

One of the first effectors recruited to PI3P is DFCP1 (double FYVE domain-containing protein 1), which binds PI3P through two FYVE domains. DFCP1 does not have an essential role in autophagy, as its depletion has no effect on autophagic flux (96); however, it is extensively used as a marker for omegasomes and phagophore-nucleation sites. Another FYVE domain-containing PI3P effector is ALFY (autophagy FYVE-linked protein). ALFY is involved in selective autophagy, where it is required for autophagic clearance of aggregates (97, 98).

### PROPPINs

Phagophore nucleation requires recruitment of the WIPIs (WD-repeat domain phosphoinositide-interacting proteins) (99, 100), which are members of the PROPPIN (β-propellers that bind phosphoinositides) family (101). The four mammalian WIPIs are seven-bladed β-propellers that bind two molecules of PI3P through FRRG (WIPI1 and -2) or LRRG (WIPI3 and -4) motifs positioned between blades five and six (102, 103). WIPI1 and WIPI2b were the first two WIPIs shown to be recruited to the omegasomes and forming phagophores (104). WIPI1 has been extensively studied (105) and is one of the two most frequently used PI3P effectors to monitor phagophore formation. Although its function is not fully understood, it is shown to be recruited to omegasomes upstream of WIPI2b (106). WIPI2b is a positive regulator of autophagy and is essential for autophagosome formation (104). It directly interacts with ATG16L1 through two arginine residues (Arg-108 and Arg-126), and this interaction is required for autophagic flux (107).

WIPI3 and WIPI4 contribute to autophagosome formation, and although both bind PI3P through LRRG motifs, it is unclear whether this binding is required for their function in autophagy (106). Depletion of WIPI4 by siRNA knockdown leads to increased LC3-II levels and accumulation of enlarged, unclosed autophagosomes (106, 108) suggesting that WIPI4 plays an important role in controlling the growth and size of autophagosomes (83, 106). Supporting this notion, WIPI4 forms a functional complex with ATG2A (106) known to act at later stages of autophagosome formation. ATG2A and ATG2B depletion increases LC3-II levels and results in the formation of enlarged LC3 puncta, suggesting that they also regulate the growth of autophagosomes (109). WIPI4 *de novo* mutations have been shown to lead to subtypes of neurodegeneration with brain iron accumulation (NBIA), such as SENDA (static encephalopathy of childhood with neurodegeneration in adulthood) (110), also known as BPAN (β-propeller protein-associated neurodegeneration) (111). In patient samples, destabilization of WIPI4 protein levels is associated with significantly reduced autophagic response (110).

## Ubiquitin-like conjugation systems

The essential event in phagophore elongation is the recruitment of Atg8 family proteins to the forming phagophore membrane, which is dependent on the ATG12∼ATG5–ATG16L1 complex. Two ubiquitin-like protein cascades are required for recruitment of the Atg8 proteins, both being highly conserved and essential for autophagy.

The Atg8 family of proteins, for simplicity referred to below as LC3 proteins, contains the following six members; LC3A, LC3B, LC3C, GABARAP, GABARAPL1, and GATE-16 (also called GABARAPL2). LC3 proteins are ubiquitin-like and are predominantly found in an unlipidated form in the cytosol. The cytosolic, unlipidated form of LC3 is referred to as LC3-I. LC3 conjugation to the headgroup of phosphatidylethanolamine (PE) on the phagophore requires several ATG proteins, which act in two ubiquitin-like cascades (for details see Ref. 112). Briefly, prior to lipidation the ATG4 protease (note there are four ATG4 family members, ATG4A–D) removes C-terminal amino acids exposing a glycine residue. This glycine is used in the first cascade for the formation of a thioester bond with a cysteine residue in the E1-like ATG7 in an ATP-dependent manner, followed by the conjugation to ATG3, an E2-like enzyme.

The ATG12∼ATG5–ATG16L1 complex, considered to have E3-like activity, is formed in the second cascade involving the ubiquitin-like ATG12, covalently conjugated to ATG5 by the E1 ATG7 and the E2 ATG10. ATG16L1 directly interacts with ATG5 and drives dimer formation of the ATG12∼ATG5 conjugate. ATG12∼ATG5–ATG16L1 is then recruited to the phagophore via WIPI2b (107). Finally, LC3 is covalently bound to the amine headgroup of PE in the membrane by interaction of ATG12 with ATG3, forming the lipidated LC3-II. LC3-II decorates both outer and inner membranes of phagophores.

Of note, the cleavage of the Atg8 family members by ATG4 and the conjugation of ATG12 to ATG5 are thought to occur during or very soon after their synthesis. The regulation of LC3 lipidation and its association with membranes to perform its essential autophagy function is driven by the production of PI3P by the PI3K complex 1 and the recruitment of WIPI2b to the phagophore (107).

## ATG9 trafficking in the process of autophagosome formation

One of the unresolved questions in the field is the source of the membranes for phagophore formation and elongation. Most studies on the formation of the phagophore membrane have, so far, revolved around ATG9. Mammalian ATG9 is a multispanning membrane protein, which consists of six highly-conserved transmembrane domains (113, 114) and is found in the Golgi complex under normal conditions. Upon amino acid starvation and autophagy induction, ATG9 disperses into peripheral compartments, including the conserved “ATG9 compartment” or “ATG9 reservoir” and recycling endosomes (114–116). During autophagy, ATG9 vesicles shuttle around the forming phagophores, interacting transiently without any detectable stable association (115). These data and other evidence have led to the suggestion that ATG9 delivers components to the forming phagophore and autophagosome (115).

ATG9 trafficking is highly dependent on the ULK1 kinase. In mammalian cells, ULK1 depletion leads to the inhibition of ATG9 trafficking from the Golgi complex (115), and as covered previously, ULK1 phosphorylation status regulates ATG9 dynamics (24). Both ATG9 trafficking and autophagy initiation are mediated in part by the phosphorylation of ATG9 by ULK1 at Ser-14 (117). Furthermore, two of the four WIPI proteins are implicated in regulation of ATG9 traffic. The depletion of WIPI2 inhibits the retrieval of ATG9 to the Golgi complex and results in the accumulation of ATG9 on omegasomes (115). Similarly, patients suffering from SENDA have increased co-localization of LC3 and ATG9, revealing WIPI4 as a potential regulator of ATG9 dynamics (110). Interestingly, increased colocalization between ATG9 and LC3 is also noted upon knockdown of ATG2A and ATG2B (109).

## Conclusions

Insight into the molecular mechanisms of autophagosome biogenesis has increased greatly in the past 10 years, but many questions remain. For example, a full understanding as to how ULK target specificity is achieved is elusive, with the identification of substrates likely to remain an area ripe for discovery. Although not covered here, the ability to derive structural information for whole complexes rather than single protein domains is providing a unique insight into the biology of autophagic signaling complexes (for more detail, see Refs. 38, 118, 119) and is likely to guide future discoveries. Nonetheless, the regulation of the activity of the signaling complex(es) remains to be fully addressed. Furthermore, the precise functions of ATG9 and the four WIPI proteins will be important to clarify in order to gain a complete mechanistic understanding of autophagosome biogenesis.

## Footnotes

2 The abbreviations used are:

ER: endoplasmic reticulum
ERGIC: ER-Golgi intermediate compartment
PI3K: phosphoinositide 3-kinase
MTOR: mechanistic target of rapamycin
AMPK: AMP-activated protein kinase
PE: phosphatidylethanolamine
PI3P: phosphatidylinositol 3-phosphate.
